# Determinants of having severe acute respiratory syndrome coronavirus 2 neutralizing antibodies in Egypt

**DOI:** 10.1111/irv.12889

**Published:** 2021-07-15

**Authors:** Amira S. El Rifay, Sara H. Mahmoud, Mohamed A. Marouf, Mokhtar R. Gomaa, Ahmed El Taweel, Noura M. Abo Shama, Mohamed GabAllah, Soha M. Abd El Dayem, Ahmed Kandeil, Ahmed Mostafa, Rabeh El‐Shesheny, Ghazi Kayali, Mohamed A. Ali

**Affiliations:** ^1^ Centre of Scientific Excellence for Influenza Viruses National Research Centre Giza Egypt; ^2^ Child Health Department National Research Centre Giza Egypt; ^3^ Paediatrics Department National Research Centre Giza Egypt; ^4^ Department of Epidemiology, Human Genetics, and Environmental Sciences University of Texas Houston Texas USA; ^5^ Life Sciences Division Human Link Dubai United Arab Emirates

**Keywords:** covariates, COVID‐19, Egypt, neutralizing antibodies, SARS‐CoV‐2, seroprevalence

## Abstract

**Background:**

Reported laboratory‐confirmed COVID‐19 cases underestimate the true burden of disease as cases without laboratory confirmation, and asymptomatic and mild cases are missed by local surveillance systems. Population‐based seroprevalence studies can provide better estimates of burden of disease by taking into account infections that were missed by surveillance systems. Additionally, little is known about the determinants of seroconversion in community settings.

**Methods:**

We conducted a cross‐sectional serologic survey among 888 participants in Egypt.

**Results:**

Neutralizing antibodies were detected in 30% of study volunteers. Age and educational level were associated with being seropositive as people older than 70 years and people with graduate degrees had lower seroprevalence. Self‐reporting cases having COVID‐19‐related symptoms such as fever, malaise, headache, dyspnea, dry cough, chest pain, diarrhea, and loss of taste or smell were all associated with having antibodies. Fever and loss of taste or smell were strong predictors with odds ratios of 2.1 (95% confidence interval: 1.3–3.5) and 4.5 (95% confidence interval: 2.6–7.8), respectively.

**Conclusions:**

Our results can guide COVID‐19 prevention and control policies and assist in determining the immunity level in some Egyptian communities.

## 1 INTRODUCTION

As of December 29, 2020, the world had more than 79 million cases of COVID‐19 of whom more than 1.7 million died.^1^ The number of cases represents laboratory‐confirmed cases and is curated by the World Health Organization (WHO) based on official reports from participating countries and territories. This figure underestimates the true burden of disease as cases without laboratory confirmation as well as asymptomatic and mild cases that are missed by local surveillance systems are not reported. Consequently, the case fatality rate (CFR), currently at 2.2% globally,^1^ may be overestimated. Population‐based seroprevalence studies can provide better estimates of burden of disease and mortality rates by taking into account infections that were missed by surveillance systems. The WHO is supporting the Solidarity II study aiming at “understanding the extent of the COVID‐19 pandemic, which in turn will allow local, national, and international decision‐makers to respond collectively to the pandemic.”

According to media reports released by the Egyptian Ministry of Health and Population, the first COVID‐19 case in Egypt was detected in a traveler from China in mid‐February 2020. Clusters of cases were not detected until March 6 after which community transmission began. The 5‐day average number of cases remained under 200 until the third week of April then increased in the last week of April as the Holy Month of Ramadan began. Towards the end of Ramadan, the 5‐day average reached 750 then exceeded 1200 in the following 2 weeks that included the Fitr Islamic Holiday. This average peaked at around 1600 cases towards mid‐June then started to gradually decrease down to around 600 cases during the third week of July. Cases dropped further over the summer months but started to increase up to around 1400 cases per day by the end of December. Overall, 131 315 cases and 7352 deaths (5.6% CFR) were reported in Egypt by December 29, 2020.^1^

Several community‐based seroprevalence studies have been conducted. In a serosurvey among blood donors in Brazil conducted in April 2020, anti‐SARS‐CoV‐2 antibodies were detected in 4% of the samples.^2^ In another population‐based study in Brazil in the period April–May 2020, the seroprevalence rate was 0.05%.^3^ In the United States, seroprevalence in Los Angeles County was estimated at 4.7% in April 2020.^4^ In a larger study involving several states during March–May 2020, calculated seroprevalence rates ranged between 1% and 6.9%.^5^ In a population‐based study in Geneva, Switzerland, 4.8% of the participants had anti‐SARS‐CoV‐2 IgG during April–May 2020.^6^ The largest seroprevalence study was conducted in Spain among more than 61 000 participants enrolled between April and May 2020. Seropositivity was around 5%.^7^ A meta‐analysis of seroprevalence studies published up to August 14, 2020, concluded that the pooled seroprevalence rate reported from 47 studies in 23 countries is 3.4%.^8^ However, very few studies assessed the determinants of seropositivity among subjects. Here, we conducted a cross‐sectional study aimed at assessing the seroprevalence and determinants of seropositivity in Egypt.

## 2 METHODS

### 2.1 Design and study population

The study population was employees of a major research institution in Egypt located in Greater Cairo, totaling around 7000.^9^ In the period August–October 2020, study personnel visited all departments of the institution on two different days and invited all individuals present at the time of the visit and who never had a laboratory‐confirmed SARS‐CoV‐2 infection to participate. Upon completing the informed consent process and signing the consent form, subjects were asked a series of demographic, health status, and COVID‐19 exposure questions using a specifically tailored questionnaire. A phlebotomist then collected a 3‐ml blood sample for serum collection. Sera were separated by centrifuging blood at 1000 *g* for 15 min. All samples were heat inactivated at 56°C for 30 min and stored at −20°C until testing.

### 2.2 Microneutralization assay

Sera were tested for neutralizing antibodies using a microneutralization assay (MN). The MN was conducted as described previously using Vero‐E6 cell monolayers.^10^ Briefly, serial twofold dilutions of heat‐inactivated sera starting with a dilution of 1:10 were mixed with equal volumes of 100 tissue culture infectious dose (TCID50 per milliliter) of hCoV‐19/Egypt/NRC‐03/2020 SARS‐CoV‐2 isolate. After 1 h of incubation at 37°C, 35 μl of the virus–plasma mixture was added in duplicate to Vero‐E6 cell monolayers in 96‐well microtiter plates. After 1 h of adsorption, the inoculums were aspirated. The plates were then incubated for three more days at 37°C in 5% CO_2_ in a humidified incubator. A virus back‐titration was performed without immune serum to assess input virus dose. Cytopathic effect (CPE) was read at 3 days post infection (dpi). The highest serum dilution that completely protected the cells from CPE was recorded as the neutralizing antibody titer. Sera testing negative at a 1:10 dilution were given a nominal value of 1:5.

### 2.3 Statistical analysis

SPSS v23 (IBM, Armonk NY) was used for analysis. Chi‐square was used to compare seropositivity rates within categorical variables. *P* value < .05 was considered statistically significant. Logistic regression was used to calculate adjusted odds ratios using all variables that were significant in bivariate analysis.

## 3 RESULTS

A total of 888 subjects participated in this study. Demographic and health data of study participants are shown in Table 1. The majority of participants were between 30 and 60 years old (83.1%) while 9.7% were <30 years and 7.2% >60 years. Females constituted 50.5% of participants. More than half of the subjects (54.7%) were college educated, 41.3% had only school education, and only 4% were not educated. The majority of the subjects (94.2%) resided in Greater Cairo composed of Cairo governorate and parts of Giza and Qalyubiya Governorates. Participants were either faculty members (27.6%), had administrative jobs (49.2%), or were support staff (23.2%). Using tobacco products was reported by 18% of the participants. A third of the participants reported having a chronic disease, and 7.8% reported using long‐term medications.

**TABLE 1:** Distribution of demographic and health data of the study

Variable	No. (%)
Age	
<30 years	86 (9.7)
31–40 years	251 (28.3)
41–50 years	223 (25.1)
51–60 years	264 (29.7)
61–70 years	51 (5.7)
>70 and above	13 (1.5)
Sex	
Female	448 (50.5)
Male	440 (49.5)
Educational level^a^	
Not educated	35 (4.0)
Elementary/intermediate	100 (11.3)
Secondary	265 (30.0)
Undergraduate	241 (27.3)
Graduate	242 (27.4)
Place of residence	
Cairo	229 (25.8)
Giza	607 (68.4)
Nile Delta	52 (5.8)
Occupation^a^	
Faculty member	242 (27.6)
Administrative	432 (49.2)
Support staff	204 (23.2)
Current tobacco user	
Yes	160 (18.0)
No	728 (82.0)
Chronic disease	
Yes	303 (34.1)
No	585 (65.9)
Long‐term medication use	
Yes	69 (7.8)
No	819 (92.2)

^a^ Totals do not add up to 888 due to missing data.

Around 39% of the participants reported having influenza‐like illness (ILI) symptoms in the 3 months preceding enrollment in the study (Table 2). Most common symptoms were malaise, sore throat, rhinitis, fever, headache, and dry cough. Fifteen percent of subjects reported being exposed to a COVID‐19 patient. This exposure was mainly at work (40.6%) or within the subject's household (34%). Among the participants, 37.8% reported attending a social gathering, and 24.2% reported domestic or international travel in the 3 months preceding enrollment. The majority of the participants (91.3%) reported using face masks; 22.9% reported using public transport. Fifty‐eight subjects (6.5%) were healthcare workers involved in direct patient care.

**TABLE 2:** COVID‐19‐related symptoms and behaviors among study participants

Variable	No. (%)
Influenza‐like illness episodes in previous 3 months	
Yes	348 (39.2)
No	540 (60.8)
Reported symptoms in previous 3 months	
Fever	143 (16.1)
Sore throat	169 (19.0)
Rhinitis	161 (18.1)
Wet cough	63 (7.1)
Dry cough	90 (10.1)
Difficulty in breathing	57 (6.4)
Chest pain	48 (5.4)
Headache	117 (13.2)
Diarrhea	68 (7.7)
Conjunctivitis	20 (2.3)
Malaise	193 (21.7)
Loss of taste or smell	94 (10.6)
Contact with COVID‐19 case in previous 3 months	
Yes	138 (15.5)
No	750 (84.5)
Place of contact with COVID‐19 case^a^	
Household	47 (34.0)
Work	56 (40.6)
Other	35 (25.4)
Attended a gathering or event in previous 3 months^b^	
Yes	336 (37.8)
No	552 (62.2)
Number of attendees at gathering or event^b^	
≤10	97 (28.9)
11–20	62 (18.5)
21–50	74 (22.0)
51–100	49 (14.6)
>100	54 (16.0)
Traveled in the previous 3 months	
Yes	215 (24.2)
No	673 (75.8)
Uses masks regularly	
Yes	811 (91.3)
No	77 (8.7)
Uses public transport	
Yes	203 (22.9)
No	685 (77.1)
Healthcare worker	
Yes	58 (6.5)
No	830 (93.5)

^a^ Among 138 subjects who reported contact with COVID‐19 case.

^b^ Among 336 subjects who attended a gathering.

**FIGURE 1:**
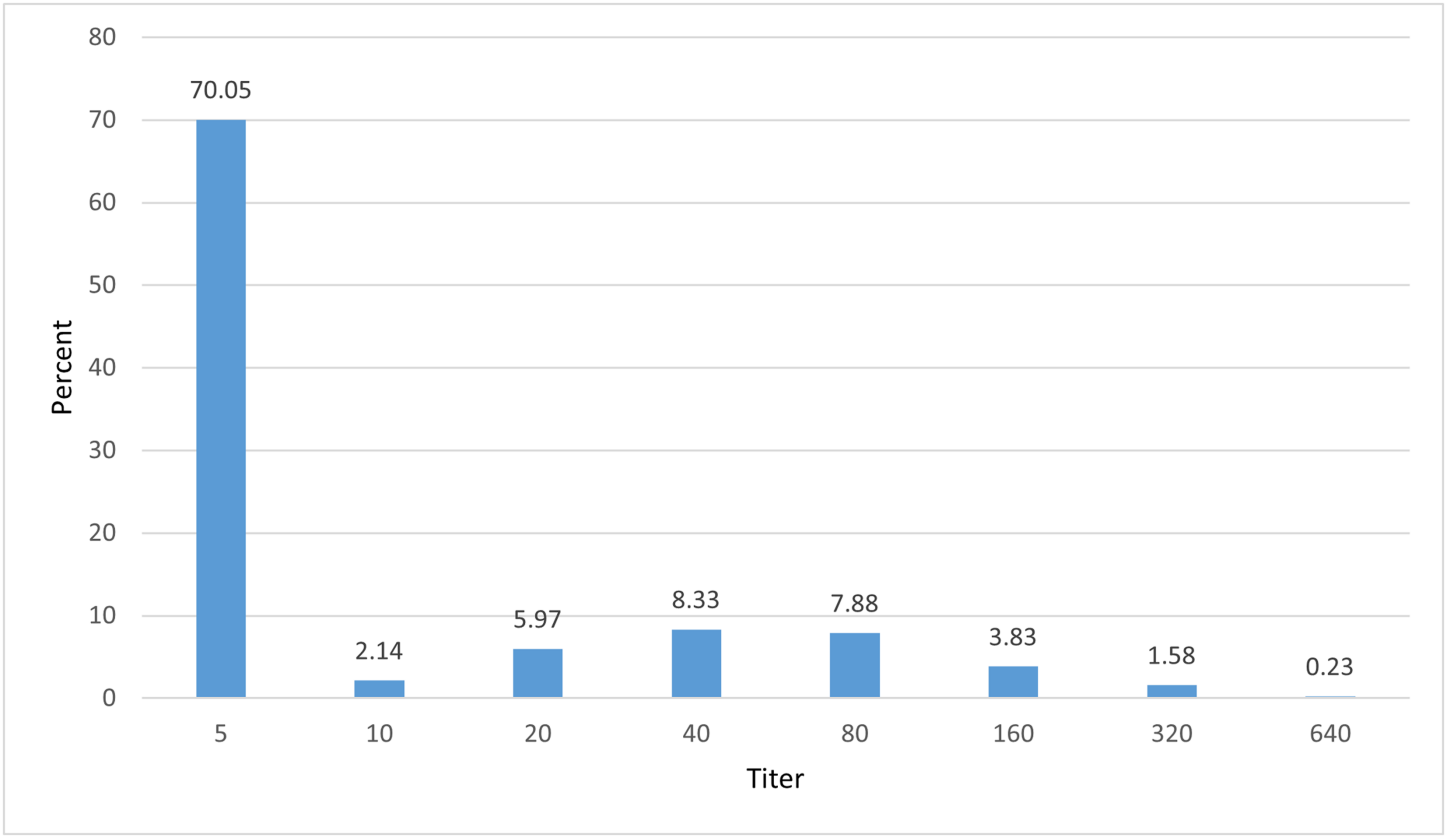
Distribution of neutralizing antibody titers among cohort participants

A total of 266 (30%) sera tested positive. Titer distribution is shown in Figure 1. Most positive sera had a titer of 1:40 (8.3%) or 1:80 (7.9%). Around 8% had a titer lower than 1:40, and around 5.6% had a titer higher than 1:80. The overall geometric mean titer (GMT) was 1:10.

Determinants of seropositivity are shown in Table 3. Age was significantly associated with antibody levels (*P* value = .039). Though most age categories had seropositivity ranging from 23.3% to 35.0%, those who were older than 70 years were seronegative. Having a graduate education or being a faculty member was protective against having antibodies against SARS‐CoV‐2. Reporting ILI was associated with seropositivity as 41.1% of those were seropositive compared with 22.8% among those who did not report ILI. Similarly, reporting fever, dry cough, difficulty breathing, chest pain, headache, diarrhea, malaise or loss of taste or smell were predictors of having an antibody titer. Odds ratios ranged from 1.6–5.6 with loss of taste or smell being the strongest predictor. None of the other collected variables were significantly associated with seropositivity. After regression analysis, fever and loss of taste or smell continued to be predictors of seropositivity. The odds ratio for fever was 2.1 (95% confidence interval 1.7–3.2) and that for loss of taste or smell was 4.5 (95% confidence interval 2.6–7.8).

**TABLE 3:** Determinants of seropositivity among study participants

Variable	Seropositive No. (%)	*P* value	Odds ratio (95% confidence interval)
Age			
<30 years	20 (23.3)	.039	‐
31–40 years	68 (27.1)		
41–50 years	78 (35.0)		
51–60 years	85 (32.2)		
61–70 years	15 (29.4)		
>70 and above	0 (0.0)		
Educational level^a^			
Not educated	11 (31.4)	<.001	‐
Elementary/intermediate	41 (41.0)		
Secondary	95 (35.8)		
Undergraduate	74 (30.7)		
Graduate	44 (18.2)		
Occupation^a^			
Faculty member	43 (17.8)	<.001	‐
Administrative	150 (34.7)		
Support staff	69 (33.8)		
Influenza‐like illness episodes in previous 3 months			
Yes	143 (41.1)	<.001	2.4 (1.8–3.2)
No	123 (22.8)		
Fever in previous 3 months			
Yes	74 (51.7)	<.001	3.1 (2.1–4.5)
No	192 (25.8)		
Dry cough in previous 3 months			
Yes	41 (45.6)	.001	2.1 (1.4–3.3)
No	225 (28.2)		
Difficulty in breathing in previous 3 months			
Yes	32 (56.1)	<.001	3.3 (1.9–5.6)
No	234 (28.2)		
Chest pain in previous 3 months			
Yes	27 (56.3)	<.001	3.2 (1.8–5.8)
No	239 (28.5)		
Headache in previous 3 months			
Yes	45 (38.5)	.031	1.6 (1.03–2.3)
No	221 (28.7)		
Diarrhea in previous 3 months			
Yes	34 (50.0)	<.001	2.5 (1.5–4.2)
No	232 (28.3)		
Malaise in previous 3 months			
Yes	86 (44.6)	<.001	2.3 (1.7–3.2)
No	180 (25.9)		
Loss of taste or smell in previous 3 months			
Yes	62 (66.0)	<.001	5.6 (3.6–8.8)
No	204 (25.7)		

a Totals do not add up to 266 due to missing data.

## 4 CONCLUSIONS

Given the wide range of clinical manifestations of COVID‐19 ranging from asymptomatic, to mild, to moderate illness requiring hospitalizing, and to critical illness requiring intensive care, the reported numbers of confirmed cases are underestimated. It is estimated that around 80% of those infected develop mild or no symptoms.^11^ Hence, population‐based seroprevalence studies become important to measure the true number of infections.^12^

Despite this, population‐based seroprevalence studies in less‐developed countries are sparse. In a serosurvey conducted in Brazzaville, Congo, in the period April–July 2020, anti‐SARS‐CoV‐2 antibodies were detected in 15% of the sampled adults.^13^ In Guilan region of Iran, 22% of study participants enrolled in April 2020 had antibodies.^14^ A larger national study in Iran conducted between April and June 2020 reported a 17% seroprevalence rate.^15^ A study among migrant workers in Kuwait revealed a 38% seroprevalence rate during May–June 2020.^16^ The seroprevalence rate among Kenyan blood donors was estimated at 4.3% during April–June 2020.^17^

In comparison with the above‐mentioned reports, our study is the only one that relied on neutralizing antibodies to determine seroprevalence of SARS‐CoV‐2 antibodies. Hence, detected antibodies are only those that directly neutralize the virus rendering it noninfectious and nonpathogenic. This means that our reported seroprevalence is lower than what would have been reported if we used assays that cover a broader range of antibody types. The overall detected seroprevalence rate is 30%, higher than those reported from other studies. If a more conservative cutoff titer of ≥1:40 is used, the seroprevalence rate becomes 22%. Despite the relatively high seroprevalence rate, none of the participants had laboratory‐confirmed COVID‐19, but a proportion of them reported having respiratory symptoms. This could potentially indicate that the majority of those who seroconverted had asymptomatic or mild disease.^18^ The reported seroprevalence may have been higher had we included individuals with previously confirmed COVID‐19.

Age was associated with seropositivity in our study as all age categories, except for those older than 70, had a seropositive proportion. Potentially, the older participants were taking more precautions to prevent infection due to the expected complications. However, caution should be applied with this interpretation as the number of individuals in the over 70 years age group was small. Age was associated with seropositivity in the Netherlands where younger adults tended to have the highest seroprevalence. But unlike our study, a proportion of the older participants had antibody titers.^19^ This discrepancy can be explained by differences in the population structure between participants in the two studies, COVID‐19 spread differences in the two countries, or different behaviors in the two populations.

Having completed graduate studies or being a faculty member was protective against being seropositive. This indicates that more educated people tend to adhere to protective measures more than others and hence are better protected against COVID‐19. Having a more senior healthcare profession was found to be protective against being seropositive in a study conducted in the United Kingdom.^20^

Self‐reporting of having respiratory symptoms was a predictor of having antibodies. A meta‐analysis of seroprevalence studies among healthcare workers showed the same finding as our study.^21^ Several self‐reported symptoms were associated with seropositivity, but the most profound was reporting of loss of taste or smell. This association remained significant even after adjusting for other variables. This finding is supported by a population‐based study in the Netherlands and by studies among healthcare workers globally.^19^, ^21^ Fever and dyspnea were also predictors of seropositivity as indicated by other studies.^19^ Anosmia and ageusia appear to be a hallmark of COVID‐19. Modifiable and behavioral variables such as wearing personal protective equipment or avoiding travel or gatherings were not statistically associated with having antibodies.

Our findings are not generalizable to the general Egyptian population due to potential selection bias. The study was conducted among employees of a single institution with a high proportion of well‐educated individuals. However, better‐educated participants had lower seroprevalence. Hence, our results may be underestimating the true prevalence in the general population. It is unlikely that this bias affected our findings related to determinants of seroprevalence. To properly estimate the seroprevalence and its determinants in the entire population, a larger national study is required. Furthermore, our findings support the notion that the number of reported COVID‐19 cases is severely underestimated due to the commonality of asymptomatic and mild disease. In contrast, Egypt reported 107 555 cases by the end of October 2020, around 0.1% of the Egyptian population. Another limitation is that the study relied on a convenience sample rather than a randomly selected sample as the institution was functioning at a reduced workforce level during the study period due to the pandemic.

In summary, our study is among the few to measure determinants of SARS‐CoV‐2 antibodies especially in a region that had a relatively less severe burden of COVID‐19. Our results can guide COVID‐19 prevention and control policies and assist in determining the immunity level in some Egyptian communities. For instance, vaccines can be directed to communities or age groups with lower immunity levels while maintaining more strict nonpharmaceutical interventions in such groups.

## AUTHOR CONTRIBUTIONS

**Amira S. El Rifay:** Conceptualization; data curation; investigation. **Sara H. Mahmoud:** Data curation; investigation. **Mohamed A. Marouf:** Data curation; investigation. **Mokhtar R. Gomaa:** Data curation; investigation. **Ahmed El Taweel:** Data curation; investigation. **Noura M. Abo Shama:** Data curation; investigation. **Mohamed GabAllah:** Data curation; investigation. **Soha M. Abd El Dayem:** Conceptualization. **Ahmed Kandeil:** Conceptualization. **Ahmed Mostafa:** Data curation; investigation. **Rabeh El‐Shesheny:** Data curation; investigation. **Ghazi Kayali:** Conceptualization; formal analysis; funding acquisition; project administration; supervision. **Mohamed A. Ali:** Conceptualization; funding acquisition; project administration; supervision.

## CONFLICT OF INTEREST

The authors declare no conflict of interest.

## ETHICS STATEMENT

Human subjects' approval was obtained from the Ethics Committee of the National Research Centre, Giza, Egypt.

## Data Availability

The data that support the findings of this study are available on request from the corresponding authors. The data are not publicly available due to privacy or ethical restrictions.
